# Common Sense Tonic Medication, with Illustrative Cases

**Published:** 1903-03

**Authors:** 


### Common Sense Tonic Medication, with Illustrative Cases.

Dr. A. W. Duvall, of Philadelphia, publishes an article in the *Medical Examiner and Practitioner*, December, 1902, with the foregoing title, in which he says, every physician of experience has many times felt the need of a satisfactory method of treatment for the exceedingly common class of cases attended with impoverishment of blood, exhaustion of nervous force and the constitutional condition designated malnutrition. It can be safely stated that the ordinarily employed tonics and reconstructives, iron, arsenic, strychnin, cod liver oil, and the like, fail in the majority of cases to effect the desired results. Reasoning *a priori* it seems that the most rational and scientific method of treating these cases is by fostering the patient's nutritive functions; and this can only be accomplished by directing treatment first to restoring the enfeebled digestive organs, so that food, the natural reconstructive and tissue builder, will be digested and assimilated. This may be said to be the first and most striking indication for treatment, inasmuch as there are always present the symptoms of atonic dyspepsia with loss of appetite, and an absolute inability on the part of the patient to take sufficient nourishment to replace the increased waste of tissue brought about by the disease processes. Most so-called tonic-nutrients and reconstructives have little or no influence upon these most important functions. Iron is administered in anemia because that element is known to be deficient in the blood; the fact that the deficiency of iron and impoverishment of the blood is but a part of a clinical condition, of which general tissue impoverishment—malnutrition—is the essential and underlying feature, seems to be disregarded. Cod liver oil, according to both scientific investigations and practical experience, is of utility only as a food fat; aside from this its therapeautic, *i.e.*, remedial value, is practically *nil.* What is most needed is a tonic, which attacks the pathologic conditions upon the rational scientific basis of restoring tone to the general system—blood, nerves, organs, nutrition, by an imitation of nature's methods of restoring waste of tissue and impoverishment of nervous force, *i.e.*, by coaxing the abrogated digestive and assimilative function—and thereby the general nutrition—to resume normal physiologic activity.

Duvall then reports a number of cases selected at random from a large number of similar ones, which were cited to illustrate the foregoing remarks upon what constitutes both rational and scientific tonic medication. The remedy selected was a mixture of gentian, taraxacum, dilute phosphoric acid, glycerine, with a small amount of sherry wine and carminatives (furnished under the name of Gray's glycerine tonic comp.), all of which ingredients have a well known and universally acknowledged tonic influence upon the digestive and assimilative functions.

The nine cases cited, of diverse natures, suffice to illustrate the statement made that if the various forms of anemia, nervous exhaustion and malnutrition—existing either independently or as a part of constitutional or organic diseases —are to be satisfactorily treated and prompt results expected, the old time so-called tonics, of which iron, arsenic, strychnin, cod liver oil, and the like, are representatives, must be supplanted by measures which are not only rational and scientific, but which have the approval of extensive and critical clinical experience.

